# The association between self-rated health and different anthropometric and body composition measures in the Chinese population

**DOI:** 10.1186/s12889-017-4249-0

**Published:** 2017-04-13

**Authors:** Kun Tang, Yingxi Zhao, Chunyan Li

**Affiliations:** 1 Department of Global Health, Peking University, Beijing, China; 2 Institute for Medical Humanities, Peking University, Beijing, China; 3 School of Public Health, Peking University, Beijing, China

**Keywords:** Self-rated Health, Obesity, BMI, Waist Circumference, Chinese

## Abstract

**Background:**

To analyze the strength of association between self-rated health and six anthropometric and body composition measures to explore the best indicator.

**Methods:**

Analyses were based on the cross-sectional data from the China Kadoorie Biobank Study and approximately 300,000 adults were analyzed. Logistics regression was used to analyze the association between self-rated health (good or poor) and anthropometric and body composition measures (height, weight, body mass index (BMI), waist circumference (WC), hip circumference (HC) and body fat percentage, waist-to-hip ratio and waist-to-height ratio). Stratified analyses were undertaken to understand the effect modification of socioeconomic status on the association.

**Result:**

Odds ratio of self-rated better health had an inverted U-shape association with weight, BMI, WC and body fat, with weight levels increasing until around 73.8 and 65.7 kg for male and female, BMI around 26.8 kg/m^2^, WC around 85.8 and 87.6 cm, body fat around 24.3 and 36.3%, and then declining thereafter. Height and HC also indicated a slightly inverted U-shape association. The strongest association was observed after adjustment was weight, with one standard deviation greater weight associated with 10.2% and 10.6% increased odds in male and female.

**Conclusions:**

Being underweight and overweight are both risk factors for poor self-rated health in males and females, and weight is the best indicator of self-rated health compared with other measures.

## Background

Self-rated health has been proved to be a valid and reliable indicator for overall morbidity [1] and a good predictor of mortality [2, 3]. Determinants of self-rated health include a great range of domains: socio-demographic factors including age [4] and employment [5, 6]; diagnosed chronic health conditions [7]; psychological factors [8]; and health behaviors [7], among which anthropometric measure of BMI has been considered an important indicator [8–15].

Past research analyzing the relation between overweight/obesity and self-rated health indicated that people with overweight and obesity reported poor self-rated health more often than those of normal weight [8–15]. However, most of those studies focused on the association with body mass index (BMI) or percentage body fat [15], and failed to include other important anthropometric and body composition measures, including height, weight, waist circumference, hip circumference, waist-to-hip ratio (WHR) and waist-to-height ratio (WHtR), some of which proved to be better indicators of body adiposity [16–18].

Moreover, little attention has been given to whether the association between these anthropometric and body composition measures and self-rated health varies across population groups of different socioeconomic status and demographic characteristics. While the research on self-rated health and anthropometric and body composition measures is limited in China, most of those studies of self-rated health focused on socioeconomic aspects [19–21].

This study aims to analyze the strength of association between self-rated health and eight anthropometric and body composition measures (i.e. height, weight, BMI, waist circumference, hip circumference, body fat, WHR and WHtR) in China to explore the best indicator, and whether the strength association varies by different socioeconomic status and demographic characteristics The result of this study could provide public health practitioners an insight of what anthropometric indicators should be used to target peoples of various socioeconomic and demographic groups to achieve maximum effectiveness and efficiency.

## Methods

### Study design and population

The present study is based on the data requested from the China Kadoorie Biobank (CKB) study, which is a prospective cohort study of chronic disease in China. Details of the study design and characteristics of the study participants have been described previously [22, 23]. Briefly, 512,891 participants without major disabilities living in administrative units (rural villages or urban residential committees), aged 30–79 years (mean age: 51.5 years), were recruited in the baseline survey from five urban (Harbin, Qingdao, Suzhou, Liuzhou, Haikou) and five rural areas (from Henan, Hunan, Sichuan, Gansu and Zhejiang) in China between 2004 and 2008. The survey sites were selected based on geographic location, population stability, quality of death and disease registries, and local commitment and capacity. Within each site, permanent residents in each of the 100–150 administrative units (rural villages or urban residential committees) that were selected for the study were identified from local records and sent a letter or leaflet inviting them to participate. The participation rate was 33% in rural areas and 27% in urban areas.

To minimize of effect of existing disease conditions and using the “healthy” population for analyses, the current study excluded 172,373 participants (73,938 males and 98,435 females) who have self-reported the following major diseases, including diabetes, coronary artery heart disease, stroke or transient ischemic attack, hypertension, rheumatic heart diseases, tuberculosis, emphysema/bronchitis, asthma, cirrhosis/chronic hepatitis, peptic ulcer, gallstone/gallbladder diseases, kidney disease, fracture, rheumatoid arthritis, psychiatric disorders, neurasthenia, head injury and cancer. Besides, the analyses also excluded people (5903 males and 7664 females) who were at the extremes of the eight anthropometric and body composition measures (i.e. >99.5 percentile or <0.5 percentile of the distribution for all eight measures) to limit effects of any possible measurement error. A total of 326,951 adults (130,418 males and 196,533 females) formed the sample for the present analyses.

Ethical approval was obtained from the University of Oxford, the China National Center for Disease Control and Prevention (CDC) and the institutional review boards at the local CDCs in the 10 regions before the start of the survey. Written informed consent was obtained from all participants.

### Measures and variables

A standardized questionnaire was administered by trained interviewers at the baseline survey using a laptop-based data-entry system, with built-in functions to prevent logical errors and missing values. Questions included socioeconomic and demographic status, health condition and medical history, behavioral pattern including smoking, drinking, physical activity and diet. In terms of physical measurements, trained staffs across the 10 survey sites conducted the standardized measurements with a protocol and instrument. All of the utilized devices were regularly maintained and calibrated for consistency in measurements.

Eight main anthropometric and body composition measures variables, either directly measured or derived, were assessed as exposure variables, including standing height, weight, body mass index (BMI), waist circumference (WC), hip circumference (HC), body fat percentage, waist-to-hip ratio (WHR) and waist-to-height ratio (WHtR). Standing height was measured to the nearest 0.1 cm with an audiometer. Weight was measured to the nearest 0.1 kg using a body composition analyzer (TANITA-TBF-300GS; Tanita Corporation), with subtraction of weight of clothing (0.5 kg in summer, 1.0 kg in spring/autumn and 2.0–2.5 kg in winter). BMI was calculated as the weight in kilograms divided by the square of the height in meters (kg/m^2^). WC and HC were measured to the nearest 0.1 cm with a soft no stretchable tape. WC was measured mid-way between the lowest rib and the iliac crest or, when this was not practicable, 1 cm above the umbilicus (usually against bare skin in both cases, but subtracting 1 cm if on top of undergarments). HC was measured at the maximum circumference around the buttocks (usually over underpants, but subtracting 1 cm if over a skirt, or 2.5 cm if over trousers). Body fat percentage was estimated by the Tanita body composition analyzer using proprietary algorithms, which reflects the fraction of total weight that was estimated to be fat weight. WHR and WHtR were calculated using the above measures. The issues of quality control (QC) was mentioned in one of the reference paper. At the baseline, QC survey data were available for 15,728 participants (3.1%), with the mean length of time between baseline and QC survey being 17 days [standard deviation (SD) = 36 days]. There was good agreement between the baseline and QC survey for several common variables. The height, weight and BMI showed extremely high correlation with baseline measures (0.99, 0.96 and 0.93, respectively), whereas for other measures of adiposity (waist and hip circumferences, and body fat percentage), they ranged from 0.82 to 0.90.

Each exposure variables were also classified into several categories for the analyses: Height was categorized into 7 groups (threshold 157, 160, 163, 166, 169 and 172 cm for male; 146, 150, 153, 156, 159, 162 cm for female), weight into 8 and 7 groups (threshold 52, 56, 60, 64, 68, 72 and 76 kg for male; 48, 52, 56, 60, 64 and 68 kg for female), BMI into 8 groups (threshold 18, 20, 22, 24, 26, 28, 30 kg/m2), WC into 8 groups (threshold 72, 76, 80, 84, 88, 92 and 96 cm for male; 66, 70, 74, 78, 82, 86 and 90 cm for female), HC into 7 groups (threshold 82, 85, 88, 91, 94 and 97 cm for male; 83, 86, 89, 92, 95 and 98 for female), body fat into 7 groups (14, 17, 20, 23, 26 and 29% for male; 23, 26, 29, 32, 35 and 38% for female), WHR into 7 groups (0.82, 0.85, 0.88, 0.91, 0.94, 0.97 for male; 0.79, 0.82, 0.85, 0.88, 0.91, 0.94 for female), WHtR into 7 groups (0.42, 0.45, 0.48, 0.51, 0.54, 0.57).

In this study, the outcome variable was self-rated health, which was an ordinal variable that rated general health on a 4-point rating scale ranging from “excellent” to “poor.” Respondents were asked, “How is your current general health status?” Responses were “excellent,” “good,” “fair,” or “poor”. For those who reported “good” and above were coded as “1”, “poor” and “fair” were coded as “0”. The dichotomies data was then included in the analyses and “0” was used as the reference group.

Other covariates included study area, sex, age category (in decile), the highest education completed (i.e. no formal school, primary school, middle school, high school, or college/university), household income last year in Chinese yuan (i.e. <2500, 2500–4999, 5000–9999, 10,000–19,999, 20,000–34,999, >35,000), marital status (i.e. married, widowed, separated/divorced, never married), smoking status (i.e. never, occasional, former, or current regular), alcohol consumption (i.e. never, occasional, former, or current regular), and total physical activity in metabolic equivalent hours per day (MET-hours/day) [24].

### Statistical analyses

Selected demographic, socioeconomic, behavioral and anthropometric characteristics were described separately for male and female. The distribution of sociodemographic, unadjusted means with standard deviations (SD) for continuous variables and unadjusted proportions for categorical variables were presented.

The association between each anthropometric and body composition measure and self-rated health was analyzed using logistics regression. Odds ratios (OR) of self-rated good health were reported within each category of the anthropometric and body composition measures, adjusting for the covariates, including study area, age category, education level, household income level, marital status, smoking status, alcohol consumption, and MET-hours/day. To analyze the association between per standard increase and the self-reporting better health, each exposure measures were divided by the SD of that particular variable and entered the regression as a continuous variable. ORs of self-rated health were regressed on levels of each anthropometric measure as a continuous variable, with adjustment for covariates as described above.

To evaluate the effect modification of socioeconomic and demographic factors, stratified analyses were performed using logistics regression, and ORs were analyzed in each stratum of socioeconomic and demographic factors. All statistical analyses were performed using SAS version 9.3 (SAS Institute Inc., Cary, North Carolina, USA), and tests results were reported significant at 0.05 levels.

## Results

Baseline participant characteristics stratified by sex are presented in Table 1. Overall, of the 326,951 participants included in the analyses, 130, 418 (39.9%) were men, and 132,297 (40.5%) resided in urban areas. At the time of the survey, the mean age was 50.74 ± 10.57 years for male and 48.85 ± 9.96 years for female. More men (58.4%) than women (45.3%) finished middle school or above. Both the prevalence of current smoking (64.2% compared with 2.1%) and weekly drinking (34.7% vs. 2.1%) were higher among men than women. Men were also more physically active (25.4 compared than women with 21.7 MET-hours/day). Compared with men, women had lower height (154.3 vs. 165.3 cm), lower weight (56.1 vs. 63.6 kg), slightly higher BMI (23.5 vs. 23.2 kg/m2) and larger HC (90.7 vs. 90.2 cm) but lower WC (78.0 vs. 81.1 cm) and higher body fat (31.6 vs. 21.7%). 71,817 male (55.1%) and 96,585 female (49.1%) reported their self-rated health as “excellent” or “good”.

**Table 1:** Basic Characteristics of 326,951 Participants by Sex

	Male (*N*=130418)	Female (*N*=196533)
Age, years (%)		
30-39	16.83	20.15
40-49	31.38	35.35
50-59	29.87	28.80
60-69	16.20	12.04
≥70	5.72	3.66
Education (%)		
Illiterate	8.65	23.37
Elementary	32.96	31.35
Middle school	33.73	27.09
High school	17.67	13.85
College and University	7.00	4.33
Household Income (%)		
<4,999 yuan	9.29	9.70
5,000-9,999 yuan	17.88	20.53
10,000-19,999 yuan	28.83	30.20
20,000-34,999 yuan	25.06	24.03
≥35,000 yuan	18.93	15.54
Marital Status (%)		
Married	93.16	90.86
Divorced, widowed, separated or never married	6.84	9.14
Tobacco use (%)		
Nonsmoker	14.01	95.68
Occasional smoker	11.47	1.69
Former smoker	10.35	0.58
Regular smoke	64.17	2.05
Alcohol use (%)		
Never regular drinker	19.00	61.78
Ex regular drinker	2.19	0.27
Occasional or seasonal drinker	33.47	33.99
Monthly drinker	6.91	1.50
Reduced intake	3.69	0.37
Weekly	34.74	2.10
Physical activity, MET-hours/day (mean ± SD)	23.53 (15.29)	21.66 (12.92)
Province (%)		
Qingdao (Urban)	8.35	6.64
Harbin (Urban)	9.03	9.54
Haikou (Urban)	5.31	6.89
Suzhou (Urban)	9.34	8.86
Liuzhou (Urban)	7.60	9.07
Sichuan (Rural)	10.74	11.54
Gansu (Rural)	10.94	11.49
Henan (Rural)	14.31	13.27
Zhejiang (Rural)	11.05	10.76
Hunan (Rural)	13.32	11.93
Anthropometric and body composition measures (mean ± SD)		
Height (cm)	165.27 (6.23)	154.33 (5.71)
Weight (kg)	63.59 (9.77)	56.12 (8.59)
BMI (kg/m^2^)	23.22 (2.97)	23.52 (3.12)
Waist Circumference (cm)	81.13 (8.90)	78.02 (8.58)
Hip Circumference (cm)	90.27 (6.26)	90.70 (6.18)
Body Fat (%)	21.72 (5.80)	31.56 (6.51)
Waist to Hip Ratio	0.90 (0.05)	0.86 (0.07)
Waist to Height Rario	0.49 (0.06)	0.51 (0.06)

**Fig. 1:**
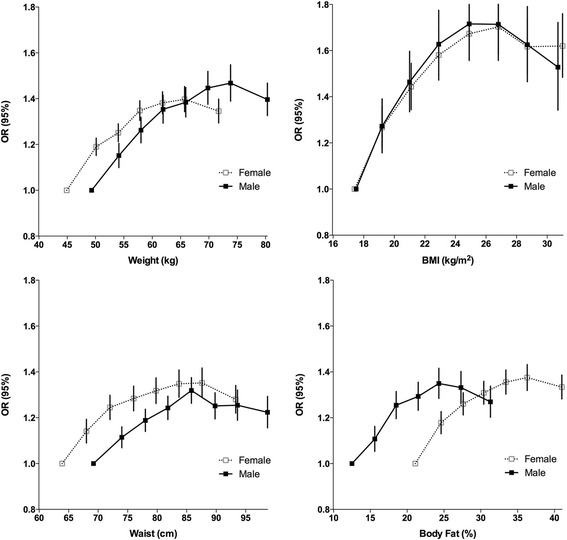
Sex-specific associations between self-rated health and 4 anthropometric and body composition measures, adjusted for study area, age category, education level, household income level, marital status, smoking status, alcohol consumption, and MET-hours/day

Figure 1 shows the sex-specific associations between self-rated health and anthropometric and body composition measures. The OR had an inverted U-shape association with weight, BMI, WC and body fat, with weight levels increasing until around 73.8 and 65.7 kg respectively for male and female, BMI level around 26.8, WC level around 85.8 and 87.6, body fat around 24.3 and 36.3, and then declining thereafter. Height, HC and WHtR also showed a slightly inverted U-shape association. There was a slightly inverted U-shape for WHR in male, but a positive linear association in female.

**Fig. 2:**
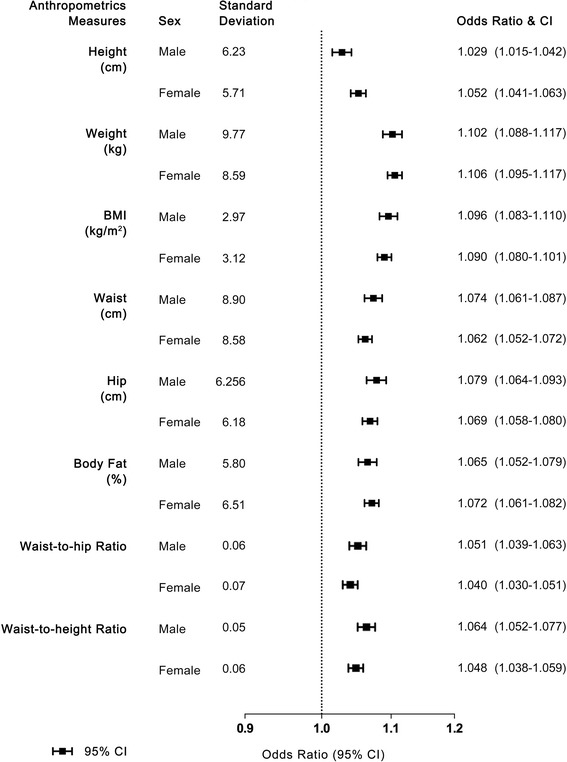
Odds ratio of self-reported good health associated with per 1 sex-specific SD of each anthropometric and body composition measure, adjusted for study area, age category, education level, household income level, marital status, smoking status, alcohol consumption, and MET-hours/day

Figures 2 shows OR of self-reported good health associated in per 1 sex-specific SD of each anthropometric and body composition measure, adjusting for other socioeconomic, demographic and behavioral covariates. In both sexes, the single strongest anthropometric indicator after adjusting for all confounders was weight, with each 1 SD greater weight associated with OR of 1.102 (95% CI 1.088–1.117) and 1.106 (95%CI 1.095–1.117) in male and female. The next strongest predictor was BMI, with OR of 1.096 (95%CI 1.080–1.110) for male and 1.090 (95%CI 1.080–1.101) for female, while height was the weakest predictors, with only 1.029 (95%CI 1.015–1.042) for male and 1.052 (95%CI 1.041–1.063) for female.

Table 2 shows the effect modification of self-rated health OR among different socioeconomic strata. Education level and household income appeared to be the strongest modifier in affecting self-rated health and anthropometric measures, while the difference of the association between self-rated health and anthropometric measures among different age group and region group (rural or urban) was weaker in significance. In our adjusted model, male with an income level between 5000 and 19,999 yuan were more likely to report better self-rated health with increase in weight than male with income above 20,000 (OR 1.131, 95%CI 1.110–1.153; vs. OR 1.080, 95% CI 1.059–1.101), while when the income level was above 20,000 yuan or education level above high school, the association between increase in anthropometric measures and increase in self-rated health was weaker. Such association was insignificant in female.

**Table 2:** Odds ratios of excellent self-rated health, for 1-SD increment in different measures in different age, income, education and region level

	Weight		BMI		Waist		Body Fat	
	Male	Female	Male	Female	Male	Female	Male	Female
Age^a^								
30–45	1.088 (1.065–1.111)	1.111 (1.094–1.129)	1.079 (1.058–1.101)	1.094 (1.077–1.111)	1.063 (1.041–1.085)	1.068 (1.051–1.085)	1.046 (1.025–1.067)	1.074 (1.057–1.090)
45–60	1.100 (1.078–1.122)	1.096 (1.080–1.112)	1.095 (1.075–1.116)	1.078 (1.062–1.093)	1.075 (1.055–1.096)	1.048 (1.033–1.063)	1.069 (1.049–1.090)	1.060 (1.045–1.075)
> 60	1.118 (1.086–1.152)	1.089 (1.062–1.117)	1.101 (1.072–1.131)	1.074 (1.049–1.100)	1.065 (1.037–1.094)	1.040 (1.016–1.064)	1.075 (1.046–1.105)	1.061 (1.037–1.086)
Income Level (yuan)^b^								
< 4999	1.139 (1.087–1.193)	1.115 (1.080–1.151)	1.153 (1.105–1.204)	1.086 (1.054–1.118)	1.132 (1.085–1.181)	1.082 (1.050–1.114)	1.113 (1.067–1.160)	1.063 (1.033–1.095)
5000–19,999	1.131 (1.110–1.153)	1.120 (1.105–1.136)	1.115 (1.095–1.135)	1.102 (1.088–1.117)	1.100 (1.081–1.120)	1.076 (1.061–1.090)	1.095 (1.076–1.115)	1.088 (1.074–1.102)
> 20,000	1.080 (1.059–1.101)	1.097 (1.079–1.114)	1.075 (1.055–1.095)	1.084 (1.067–1.101)	1.050 (1.031–1.070)	1.054 (1.037–1.071)	1.034 (1.015–1.053)	1.059 (1.043–1.076)
Education Level^c^								
Under Primary school	1.126 (1.072–1.183)	1.100 (1.078–1.123)	1.112 (1.062–1.164)	1.095 (1.074–1.117)	1.073 (1.026–1.121)	1.070 (1.049–1.090)	1.069 (1.023–1.116)	1.072 (1.052–1.093)
Middle & High School	1.106 (1.090–1.122)	1.103 (1.090–1.116)	1.099 (1.085–1.114)	1.082 (1.070–1.094)	1.079 (1.065–1.093)	1.052 (1.040–1.064)	1.071 (1.057–1.086)	1.064 (1.053–1.076)
College & University	1.047 (0.999–1.098)	1.076 (1.022–1.133)	1.029 (0.983–1.077)	1.041 (0.989–1.095)	1.004 (0.957–1.054)	1.046 (0.990–1.104)	0.983 (0.936–1.031)	1.034 (0.982–1.089)
Region^d^								
Rural	1.102 (1.083–1.122)	1.104 (1.090–1.118)	1.102 (1.084–1.120)	1.088 (1.074–1.101)	1.080 (1.063–1.097)	1.071 (1.058–1.084)	1.074 (1.058–1.091)	1.073 (1.060–1.086)
Urban	1.100 (1.078–1.122)	1.099 (1.083–1.117)	1.083 (1.062–1.103)	1.083 (1.067–1.100)	1.063 (1.042–1.084)	1.037 (1.021–1.053)	1.046 (1.025–1.067)	1.059 (1.042–1.075)

^a^ adjusted for study area, sex, the highest education completed, household income last year in Chinese yuan, marital status, smoking status, alcohol consumption, and total physical activity in metabolic equivalent hours per day

^b^adjusted for study area, age category sex, the highest education completed, marital status, smoking status, alcohol consumption, and total physical activity in metabolic equivalent hours per day

^c^adjusted for study area, age category sex, household income last year in Chinese yuan, marital status, smoking status, alcohol consumption, and total physical activity in metabolic equivalent hours per day

^d^adjusted for age category sex, the highest education completed, household income last year in Chinese yuan, marital status, smoking status, alcohol consumption, and total physical activity in metabolic equivalent hours per day

## Discussion

This study is the first and also the largest scale research in China concerning the strength of association between self-rated health and anthropometric and body composition measures. One of the major findings was that weight was most strongly associated with self-reporting good health, while BMI was also a good indicator. Further analyses also indicated that the association could be modified by sex, income and education level. The association between self-rated health and anthropometric and body composition measures appeared relatively stronger among male with less income and lower education, while weaker among female.

There are several potential limitations of the present study: firstly, in the present analysis, we excluded patients with all major chronic preconditions, which covered a wide range of 19 diseases (e.g. cardiovascular disease, diabetes, hypertension, etc.). Nonetheless we only excluded the participants with self-reported diseases, and the population may not be regarded as completely “healthy”. . Secondly, the study used Tanita body composition analyzer, which was originally developed and validated only in Japanese populations. Although little evidence suggests there’s significant difference among East Asian populations like the Chinese and Japanese, in terms of body fat composition. It could potentially create a systematic bias in the analysis. Thirdly, the study population was selected from the baseline data on a cohort study and the healthy volunteer effect may exist. The population might be more sensitive to health issues and more likely to report bad health. However, the large sample size and excluding patients with major diseases shall reduce the effect to minimal. Lastly, the participation rate of the study is relatively low. While the low response rate is a problem for population based prevalence study, our study, which is drawn from the baseline of a large cohort study, does not mean to represent the whole Chinese population. Moreover, like in the U.S. Nurses’ Health Study [25] and the Health Professionals Study [26], a particular study population with strong homogeneity could already generate important associations with strong clinical and public health implications.

The inverted U-shaped associations between the four anthropometric and body composition measures (weight, BMI, WC and body fat) and self-rated health found in this study indicated that both overweight and underweight were associated with poor self-rated health. Being underweight (lower weight, BMI, body fat and WC) might be considered malnourished which indicates bad health in Chinese people’s traditional opinions, while the stigma of obesity is growing globally as obesity rates rise, thus weight, BMI, WC and body fat being too high might also give people indirect effect of obesity in limiting their functional capacity, and also an ill feeling mentally. This is the first analysis of the association between BMI and self-rated health in China, and the findings were consistent with those previously reported by several smaller studies [9, 11, 14, 27] but differ from those in several other studies, which indicated that only being overweight was associated with poor self-rated health [28], or in some cases did not discuss underweight [12, 15]. The reasons for these inconsistencies are uncertain, but this study is larger than any previous studies. One possible explanation is that all the participants in the study are middle-aged to elderly Chinese, and there might be possible societal or cultural differences regarding body image and health with other population [13]. Moreover, unlike most former studies, our main analyses also excluded participants with major diseases, which enable us to explore the unbiased association between anthropometric and body composition measures and self-rated health.

Our findings suggested a positive linear association between WHR and self-rated health in females but not males. This is different to the associations with other anthropometric indices. However, whether the same result can be observed in other studies of the Chinese population still needs to be validated. Further more, if such observation is indeed true, further studies should be conducted to understand the underlying physiological or psychological mechanisms.

Our study also found that weight and BMI are better indicators than WC and WHR, suggesting that general adiposity might be a better indicator of self-rated health than central or regional adiposity. The 1-SD increase of weight and WC could be considered similar effect on adiposity, as past studies have established that a 3 kg increase in weight was corresponded to approximately 3 cm increase in WC [29], indicating that the superiority of weight and BMI to WC is due to their direct effect on self-rated health. One possible explanation could be that people are more sensitive to change in weight than WC, as weight is easier for daily measurement and WC is a variable that seldom used in daily life.

In the further analyses, we found that the strength of association between self-rated health and anthropometric and body composition measures differs among socioeconomic status especially education level and household income. While middle-income and lower educated male tended to have the strongest influence with increase in adiposity to increase in self-rated health, one possible explanation is that the group of higher education level and income level has overall better self-rated health as explained in several former studies [28]. This group with higher education level has more access to health knowledge and better health seeking behavior, thus is less influenced by anthropometric and body composition measures. The population with lower education level and less household income has poorer self-rated health on average, and is more strongly affected by change in the measures. Our study also found that the association was weaker among female, and this may be due to the fact that almost all female face greater societal pressures, such as weight discrimination and body image problems [30, 31], regardless of socio-economic status.

## Conclusion

The current study describes the strength of association between self-rated health and anthropometric and body composition measures. It demonstrates that being underweight and overweight are both risk factors for poor self-rated health in males and females in the Chinese populations. Further analyses also indicate that general adiposity is better associated with self-rated health than central adiposity, and the association between self-rated health and anthropometric and body composition measures are modified by sex, income and education level. Additional research is needed to study other anthropometric and adiposity measures’ strength of influence on self-rated health, including weight-hip ratio, height-adjusted weight.

Our study findings have strong implications for public health interventions. Given the strong association between self-rated health with BMI and weight, these measures could be a better option as the indicators to improve the self-perceptions of health among specific social and economic subgroups of the Chinese populations, hence maximize the effectiveness of public health interventions towards obesity, diabetes and other metabolic diseases.
